# Camel milk ameliorates inflammatory mechanisms in an alcohol-induced liver injury mouse model

**DOI:** 10.1038/s41598-021-02357-1

**Published:** 2021-11-24

**Authors:** Liang Ming, Bule Qi, Shiqi Hao, Rimutu Ji

**Affiliations:** 1 Key Laboratory of Dairy Biotechnology and Bioengineering, Ministry of Education, College of Food Science and Engineering, Inner Mongolia Agricultural University, Hohhot, 010018 Inner Mongolia China; 2 Camel Research Institute of Inner Mongolia, Alashan, 737300 Inner Mongolia China

**Keywords:** Molecular biology, Diseases

## Abstract

Camel milk (CM) is considered to protect the liver in the practice of traditional medicine in nomadic areas. The purpose of the present study was to investigate the effects of CM on the hepatic biochemical and multiple omics alterations induced by chronic alcoholic liver disease (ALD). An intragastric gavage mice Lieber DeCarli + Gao binge model (NIAAA model) was employed to investigate the inflammatory mechanism of camel milk on the liver tissue of mice. A gut microbiota of the feces of mice and transcriptomic and proteomic analyses of the liver of mice were performed. Analysis of serum and liver biochemical indexes revealed that camel milk not only prevents alcohol-induced colonic dysfunction and lipid accumulation, but also regulates oxidative stress and inflammatory cytokine production to protect against chronic ALD in mouse. The gut microbial community of mice treated with camel milk was more similar to the untreated control group than to the model group, indicating that the intake of camel milk pre- and post-alcohol gavage effectively prevents and alleviates the intestinal microbial disorder caused by chronic alcoholism in mice. Furthermore, the results of the transcriptomic and proteomic analyses of the liver tissue showed that camel milk can improve alcoholic liver injury in mice by regulating inflammatory factors and immune system disruptions. This study provides insights into the molecular mechanism by which camel milk can be developed as a potential functional food with no side effects and against liver injury.

## Introduction

Liver is the well-known main organ of alcohol metabolism. The amount of alcohol metabolized by liver accounts for 90–98% of the total intake of human body. Therefore, excessive alcohol consumption will cause serious damage to the liver. Currently, there are approximately 2 billion people drinking alcohol all over the world, and more than 75 million identified as alcohol use disorders and are at risk of alcohol-related liver diseases^1^. Globally, cirrhosis and liver cancer lead to 1.16 million and 788,000 deaths, respectively, making them the 11th and 16th most common causes of death each year^2^, which brings an important financial burden to the medical system. Chronic and excessive alcohol consumption produces alcoholic liver disease (ALD), in which long-term consumption and higher doses of alcohol are important factors^3^. ALD is a complex process, including a wide spectrum of hepatic lesions, the most characteristic of which are steatosis, hepatitis, and fibrosis/cirrhosis^1,4^, causing liver failure or liver cancer in the final stages, leading to death. Excessive accumulation of alcohol results in various morphological, functional abnormalities of liver tissue, oxidative stress and hepatocyte hypoxia, as well as the induction of immune response^5,6^.

The pathogenesis of ALD is known to be complex, mainly including direct damage to hepatocytes by ethanol and its toxic metabolites, increase of reactive oxygen species (ROS) and/or decrease of antioxidant level, accumulation of fat in hepatocytes, Kupffer cell induced inflammation^4^. In addition, the lipid peroxidation induced by chronic alcohol intake can produce malondialdehyde and 4-hydroxynonenal, which lead to formation of protein adducts and cause adaptive immunity as antigens^7^. Immune function is paradoxically enhanced by inflammation^7^. Furthermore, the gut microbiota is an important target and participant of gut barrier alteration upon alcohol consumption^8^. Bacterial-derived LPS is increased in the hepatic circulatory system of the liver due to mucosal damage, gut microbiota dysbiosis, and intestinal permeability. This promotes interaction between LPS and Kupffer cells’ toll-like receptor 4 TLR4, leading to the release of pro-inflammatory cytokines (e.g., tumor necrosis factor a (TNF-a) and IL-1β) and resulting in inflammatory infiltration and fibrosis in the liver^9^.

At present, however, there are no specific drugs or therapy available to reverse the progress of ALD in humans, and abstinence from alcohol is the most effective and non-toxic treatment for ALD. However, with the deepening of research, nutritional strategies targeting experimental ALD, such as anti-inflammatory, antioxidant, and immune system-regulating nutrients, are of growing interest as promising approaches showing fewer side effects than synthetic drugs^10^. Related studies support that livestock milk has beneficial effects on the prevention and treatment of numerous illnesses, including ALD^11,12^. For instance, camel milk (CM) is considered to have hepatoprotective^11,12^ and anti-diabetic effects^13,14^, as well as provides protection against asthma, tuberculosis, and edema^15^. However, the potential hepatoprotective effects of camel milk on chronic alcohol-induced lesions have not been investigated thus far. Therefore, in the present study, a mouse liver injury model induced by long-term alcohol was established to explore the protective effect of camel milk on chronic liver injury caused by alcohol intake and the signal mechanism of inflammatory response.

## Results

### Camel milk improves alcohol-induced serum aminotransferase, oxidative stress, and inflammation in the liver

**Figure 1:**
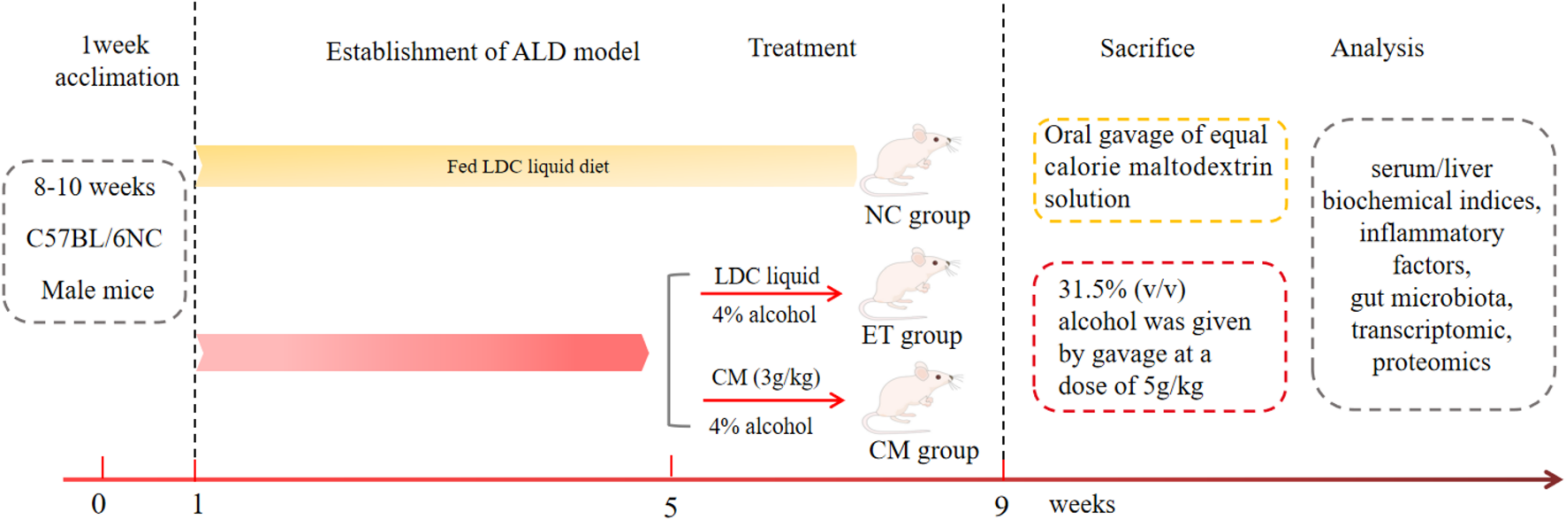
The schedule of the experimental protocol and drug administration. *NC* control group, *ET* ethanol-containing Lieber–DeCarli liquid diet group, *CM* camel milk group. *LDC* Lieber–DeCarli, *CM* camel milk.

**Figure 2:**
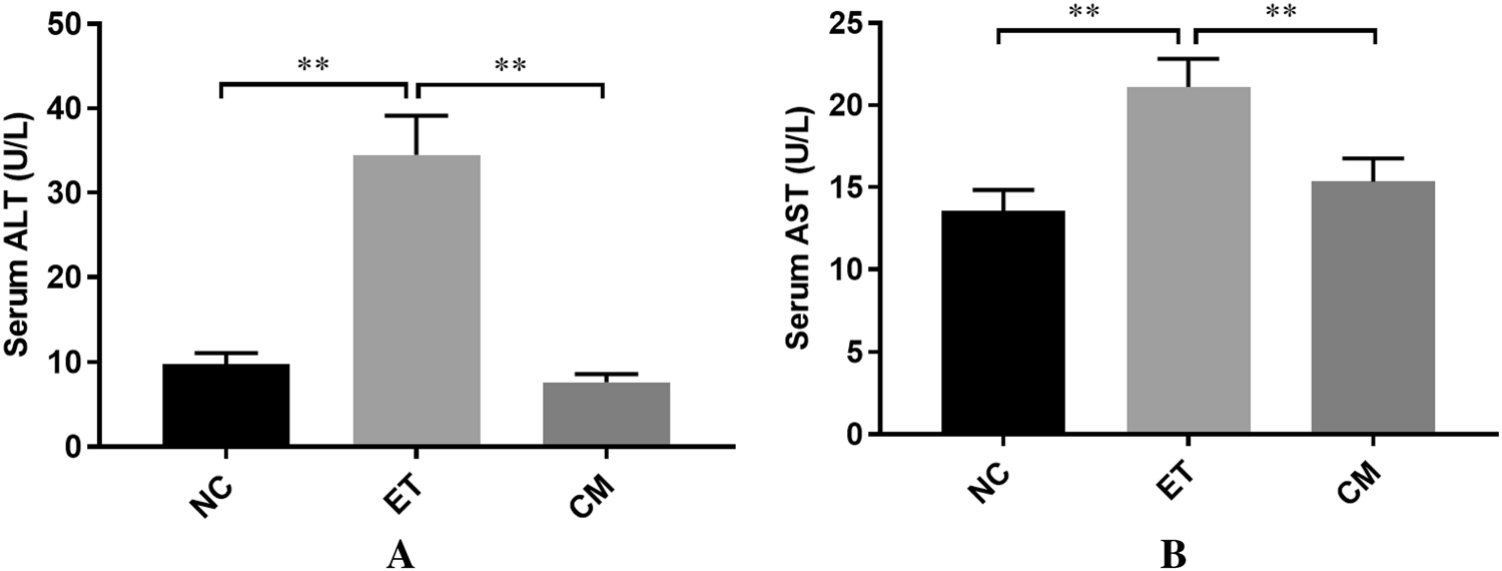
Effects of CM on the serum levels of ALT (**A**) and AST (**B**). *NC* control group, *ET* ethanol-containing Lieber–DeCarli liquid diet group, *CM* camel milk group. Significantly different showed *p < 0. 05 and **p < 0.001.

A complete scheme of the Lieber DeCarli + Gao binge model (NIAAA model) is shown in Fig. 1. To evaluate whether camel milk (CM) protects against liver injury by an LDC liquid diet, the levels of serum ALT and AST were measured in this study. Compared to the NC group, the ET group displayed a significant increase in ALT and AST, while the CM group demonstrated an obvious decrease (*p* < 0.001) (Fig. 2A,B), which initially indicates that camel milk protects against chronic alcoholic liver damage and could relieve damaged tissue. Furthermore, the marker of lipid peroxidation MDA activity in the liver tissue of the ET group was significantly higher than that in the NC group. Compared to the NC group, the antioxidant enzyme activities of SOD and GSH were decreased in the ET group, although this did not reach the level of statistical significance (*p* > 0.05) (Table S1). Furthermore, camel milk also inhibited the alcohol-induced production of TNF-a, IL-6, and IL-1β and enhanced the level of IL-10 (Table 1). All of these results suggest that camel milk not only decrease alcohol-induced aminotransferase, but also regulates oxidative stress and inflammatory cytokine production to protect against chronic ALD in mice.

**Table 1:** Effects of CM on the levels of TNF-a, IL-6, IL-1β, and IL-10 in mouse liver.

Group	TNF-a (pg/mL)	IL-6 (pg/mL)	IL-1β (pg/mL)	IL-10 (pg/mL)
NC	651.09 ± 10.19	122.13 ± 1.90	134.06 ± 2.15	434.87 ± 10.37
ET	745.49 ± 7.08**	132.15 ± 1.01**	148.03 ± 1.93**	405.513 ± 8.47
CM	627.07 ± 4.34^##^	124.84 ± 1.78^#^	137.31 ± 1.87^##^	533.97 ± 5.56^##^

Data represent the mean ± standard error of the mean (n = 8 mice).

Significantly different from the control group at *p < 0. 05 and **p < 0.001; Significantly different from the ET group at ^#^*p* < 0. 05 and ^##^*p* < 0.001.

### Camel milk affects alcohol-induced LPS and TG

**Figure 3:**
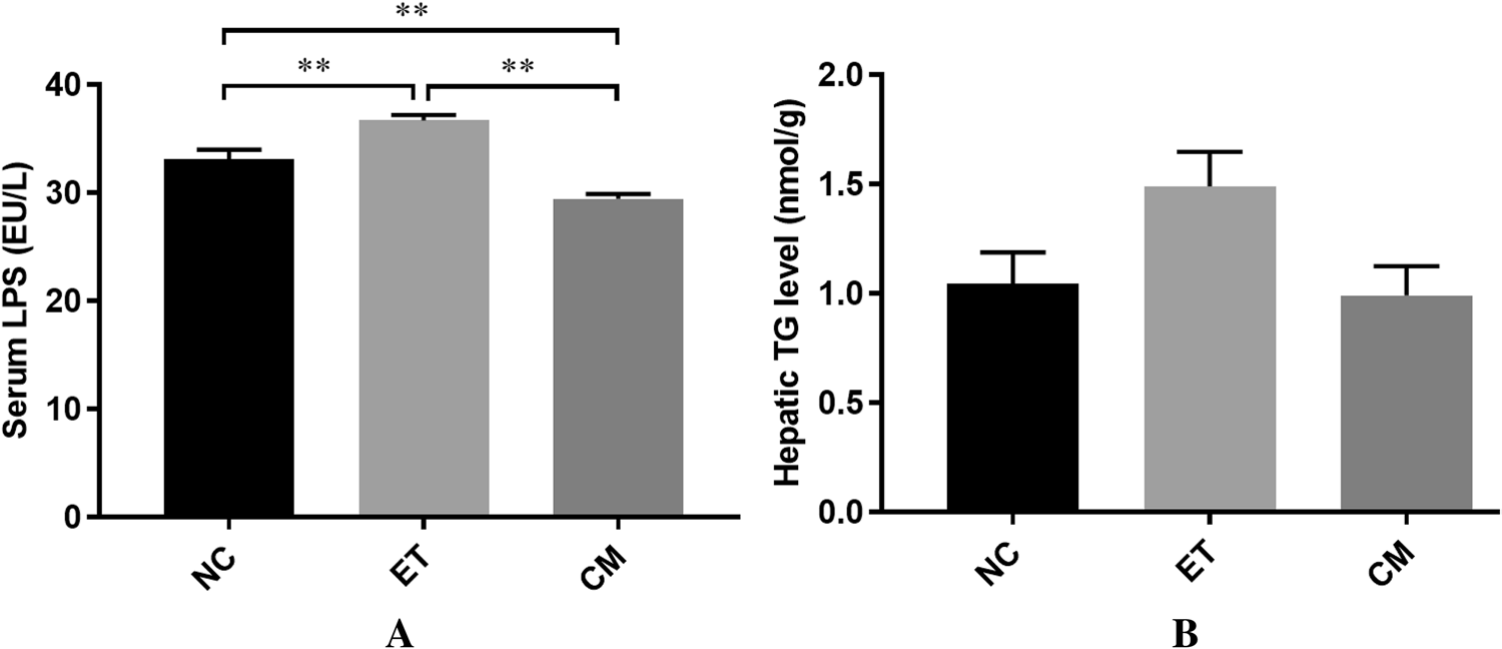
Effects of CM on the serum levels of LPS (**A**) and hepatic of TG level (**B**). *NC* control group, *ET* ethanol-containing Lieber–DeCarli liquid diet group, *CM* camel milk group. Significantly different showed *p < 0. 05 and **p < 0.001.

As shown in Fig. 3, the level of LPS was markedly increased in the ET group, while significantly decreased in the CM group compared to the NC group (*p* < 0.001) (Fig. 3A). This implies that alcohol induces intestinal mucosal injury in mice, which can cause quantitative and qualitative changes in intestinal flora and can increase intestinal permeability^16^. Compared to the NC group, the content of TG in the hepatic liver of the ET group increased, indicating that there was a large amount of lipid accumulation in the liver cells. Meanwhile, the decrease in TG content in the CM group suggests that camel milk significantly inhibits an increase in the accumulation of liver triglycerides induced by chronic alcohol, but none of these results reached a significant level (Fig. 3B). The above analysis shows that camel milk could prevent alcohol-induced colonic dysfunction and lipid accumulation, and then inhibits the increase in LPS and TG content.

### Camel milk induces changes in microbial diversity and populations

**Figure 4:**
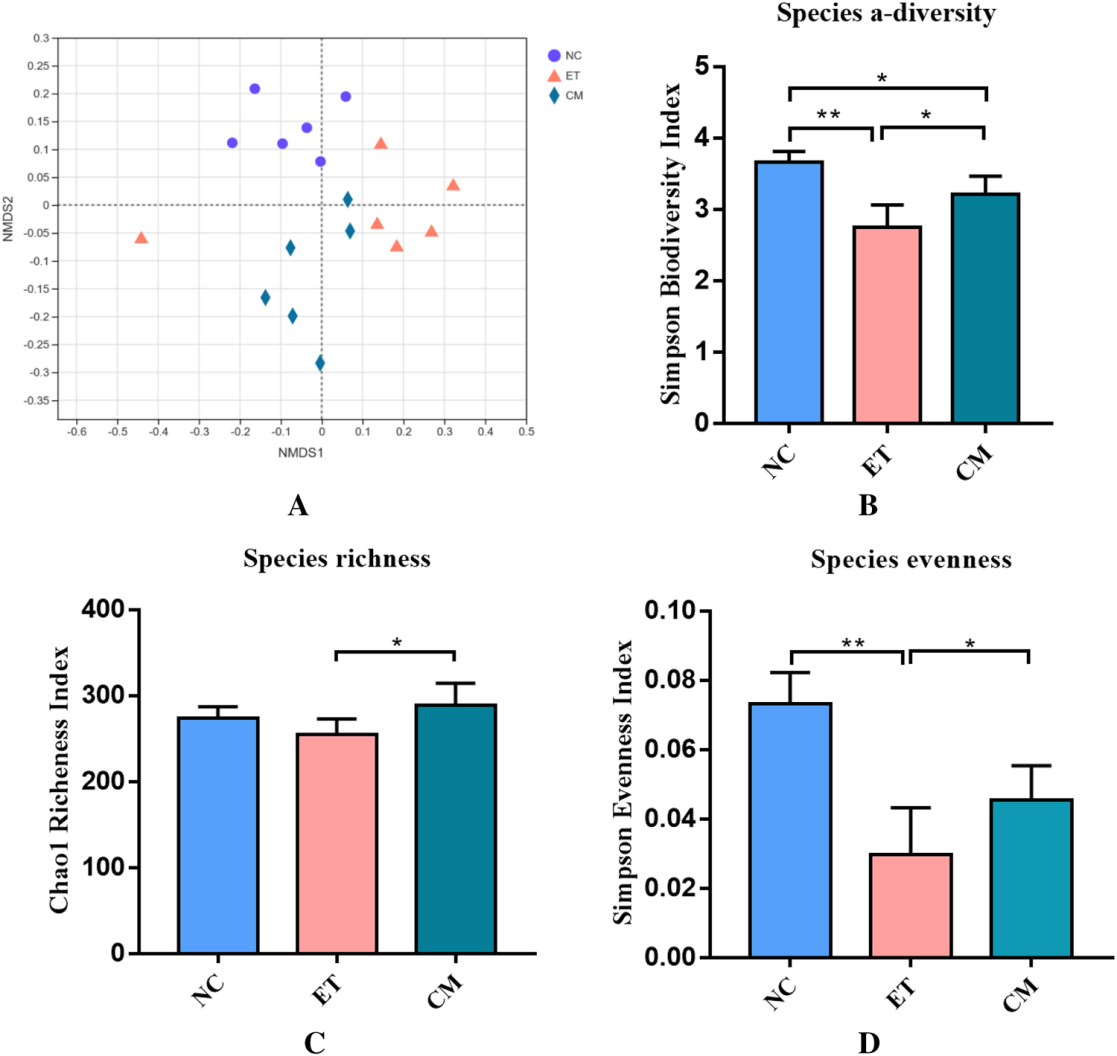
Spatial ordination and bacterial diversity deduced by 16S profiling. (**A**) Nonmetric dimensional scaling of the three mice groups; (**B**) bacterial diversity (Simpson Biodiversity Index); (**C**) bacterial richness (Chao1 Richness Index); (**D**) bacterial evenness (deduced from Simpson Index). *NC* control group, *ET* ethanol-containing Lieber–DeCarli liquid diet group, *CM* camel milk group.

The gut microbiota is recognized as a potential contributing factor to the development of ALD in mice. The differences in the intestinal microbiota among the NC, ET, and CM groups were investigated (Table S2). Meanwhile, based on non-metric dimensional scaling established by the distance matrix at the species taxonomic level, and the differences within the intestinal microbial population structure between the groups were visualized (Fig. 4A). The results of the beta diversity of gut microbiota showed a distinct cluster for each group, indicating that the difference within the group was small and that the microbial community structure was similar (Fig. 4A). Notably, the camel milk-treated mice exhibited a significantly (*p* < 0.05) increased cecal microbial a-diversity compared to the alcohol-treated mice (Fig. 4B–D).

When comparing the relative abundance of bacterial taxa between treatment groups, we observed a significant phylum-wide shift from *Firmicutes* to *Bacteroidetes* upon camel milk supplementation (Fig. S1). At the genus level, the abundance of *Ruminococcaceae_UCG-013* significantly decreased (*p* < 0.05), while the abundance of *norank_f__Muribaculaceae* and *Alloprevotella* significantly increased (*p* < 0.05) due to the camel milk challenge (Fig. S2). Compared to the ET group, the proportion of *Ruminococcaceae_UCG-013* significantly decreased (*p* < 0.001), whereas the proportions of *Muribaculaceae* and *Lachnospiraceae* significantly increased (*p* < 0.05) in the NC and CM groups (Fig. S3A,B). *Blautia* and *Mucispirillum* significantly decreased, while *Romboutsia* significantly decreased in the ET group compared to the control group (*p* < 0.05).

### Effects of chronic alcohol-induced liver injury on the liver transcriptome

After removing the low-quality reads and conducting quality control, a total of 490.20 ± 19.77, 579.19 ± 10.05, and 546.78 ± 22.33 million clean reads were obtained for the CM, ET, and NC groups, respectively (Table S3). The clean GC content of each group ranged from 49.22 to 50.06%, and the value of Q30 ranged from 96.44 to 96.74%. To evaluate the quality of the RNA-Seq data, the total clean reads were mapped to the reference genome (Mus_musculus, GRCm38.p6, http://asia.ensembl.org/Mus_musculus/Info/Index). A high proportion of the clean reads were mapped to the mouse reference genome using Tophat2 (http://tophat.cbcb.umd.edu/), that is, 95.53% from NC, 94.14% from ET, and 94.95% from CM. Additionally, of the total mapped reads, roughly 87.38% in each group corresponded to exons. Together, all of the results indicate that the RNA-Seq data were reliable.

**Figure 5:**
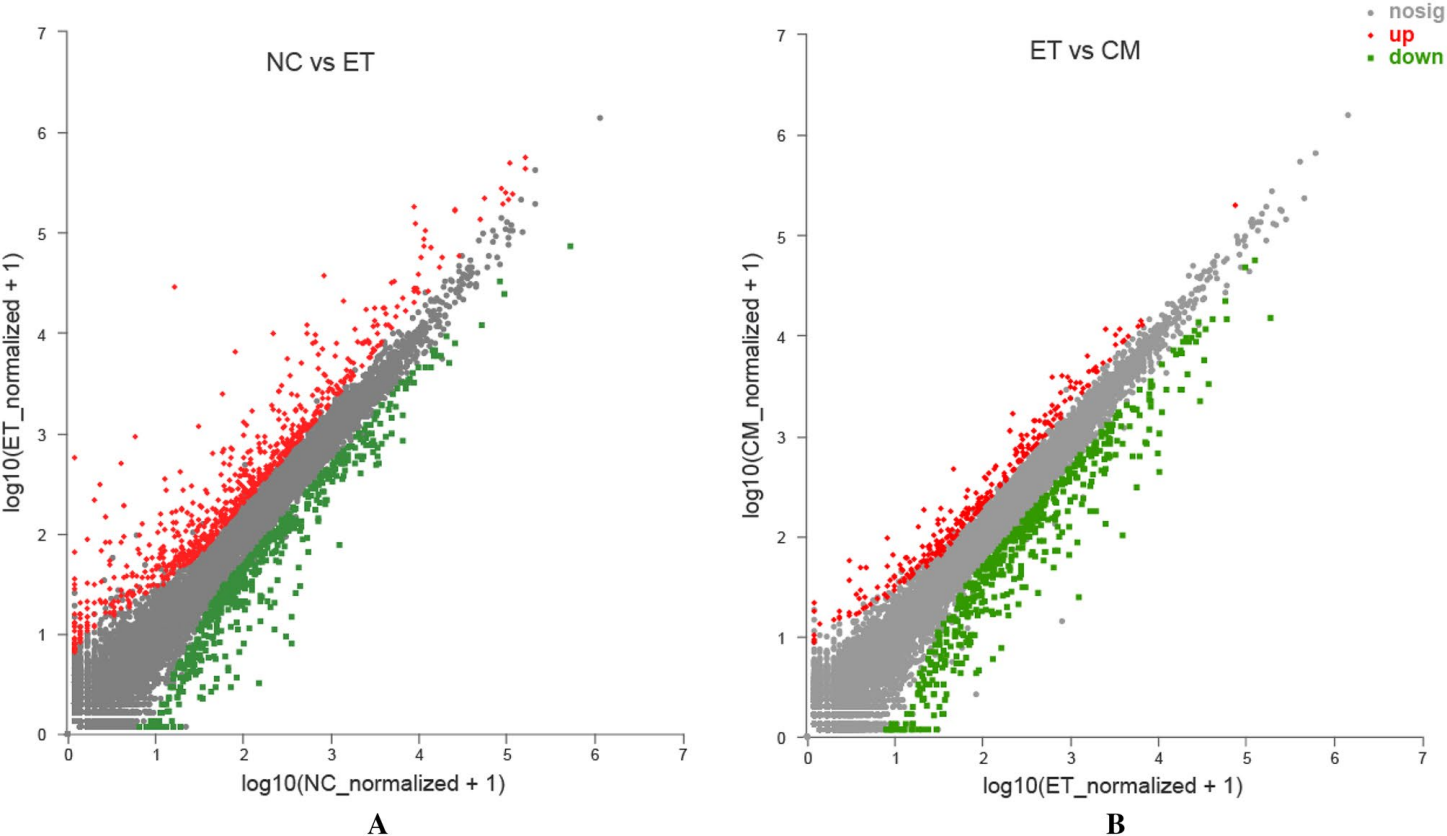
Scatter plot displaying DEGs between NC vs ET (**A**), and ET vs CM (**B**). Up-regulated and down-regulated genes are shown in red and green, respectively. Black dots represent genes with similar expression levels. *NC* control group, *ET* ethanol-containing Lieber–DeCarli liquid diet group, *CM* camel milk group.

The gene expression levels in the NC, ET, and CM groups were evaluated by counting the number of mapped reads per gene, as presented in a Venn diagram in Fig. S4. A total of 10,634 differentially expressed genes (DEGs) were commonly expressed in all three groups, whereas 402, 247, and 185 DEGs were expressed only in the NC, ET, and CM groups, respectively. Through analysis of the DEGs, we identified 699 DEGs in the ET and CM groups (249 upregulated and 450 downregulated) and 1158 DEGs in the ET and NC groups (611 upregulated and 547 downregulated; Fig. 5, Tables S4-1, -2).

### Gene annotation and functional enrichment analysis

To elucidate the potential mechanisms of CM involved in the protective effect against chronic alcohol disease, we performed GO functional and KEGG enrichment analyses on pairs of comparison groups (NC versus ET and ET versus CM) (Table S5). In the previous study, we found that CM can adjust the inflammatory reaction to ameliorate alcoholic liver injury^12^. In present study, we also observed representative KEGG pathways that contained inflammatory response signaling pathways, such as inflammatory mediator regulation of TRP channels and IL-17 signaling pathway, and all of these reached a significant level (NC versus ET and ET versus CM; *p* < 0.05). In addition, immune system-related signaling pathways, such as the Toll-like receptor signaling pathway, the NOD-like receptor signaling pathway, and Th17 cell differentiation, were identified, although these did not reach a significant level (NC versus ET and ET versus CM; *p* > 0.05). Compared to the NC group, the DEGs related to the inflammatory (Plcb4, Cyp2c70, Cyp2c55, Il1r1, Adcy1, Traf3ip2, Ikbkg, and Ikbke) and immune (Ikbkg, CD14, Ikbke, Plcb4, Ikbkg, Nrp12, Hsp90aa1, Ikbke; Il1r1, Il12rb1, and Il6ra) systems were upregulated in the ET group, while downregulated in the CM group (Fig. S5). This indicates that chronic alcohol consumption increases the expression of inflammatory factors and disrupts the immune system in complex ways. However, CM administration could ameliorate these disruptions.

### LC–MS/MS analysis

**Figure 6:**
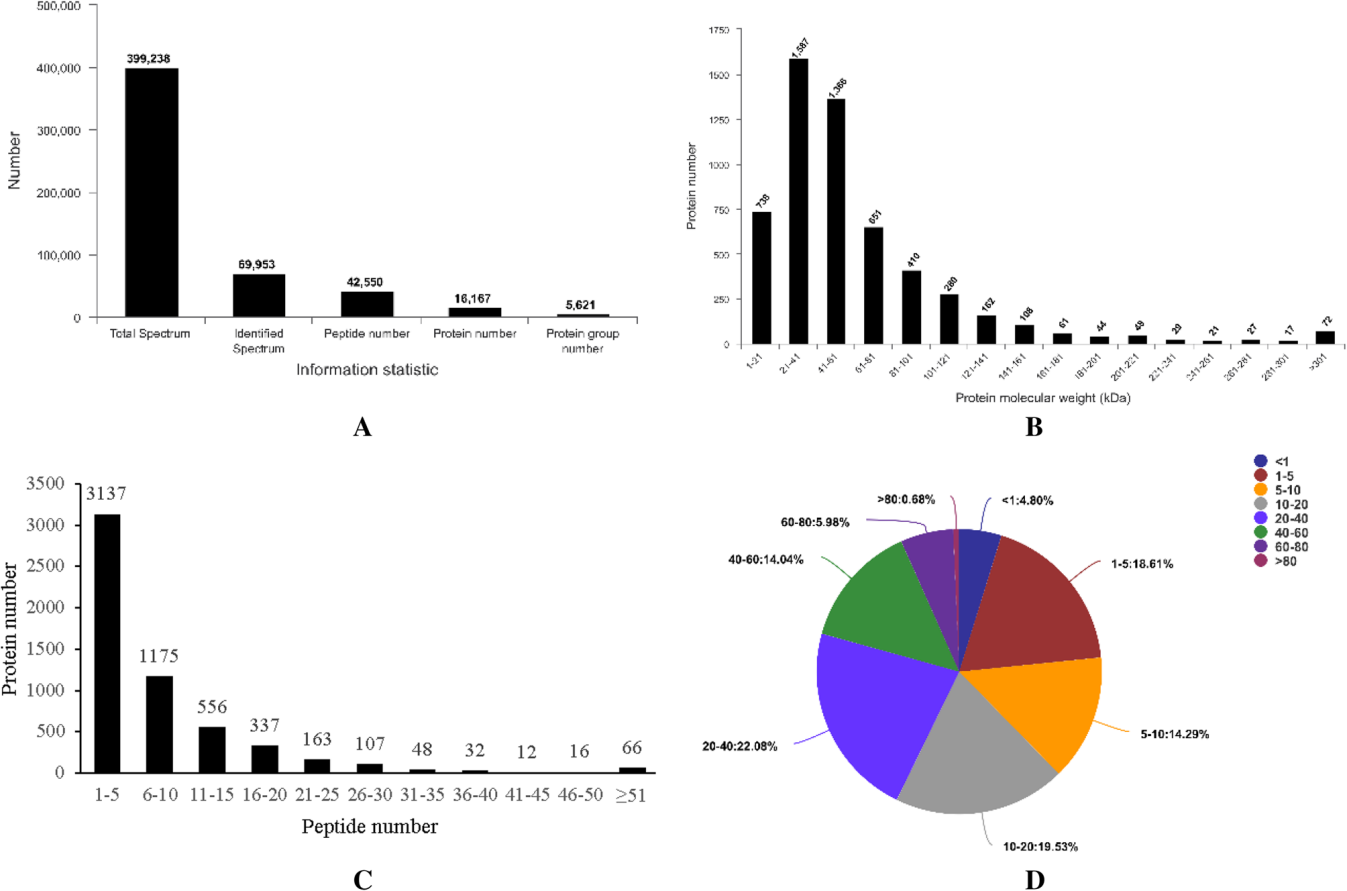
Overview of protein identification information. (**A**) Basic information on protein identification. (**B**) Distribution of the proteins identified according to molecular weight. (**C**) Protein coverage by the peptides identified. (**D**) Distribution of proteins containing different numbers of identified peptides. *NC* control group, *ET* ethanol-containing Lieber–DeCarli liquid diet group, *CM* camel milk group.

A total of 399,238 spectra were obtained from the 10PLEX liquid chromatography–tandem mass spectrometry (LC–MS/MS) analysis, as well as 42,550 peptides, which corresponded to 16,167 proteins were detected (Fig. 6A). The protein molecular weight was mainly distributed between 1 and 100 kDa (84.54%) (Fig. 6B), and 55.53% of the proteins were represented by one to five peptides each (Fig. 6C). The majority of the proteins were identified with a high peptide coverage (Fig. 6D), indicating that good sequence coverage of the proteins was determined.

### Identification and functional analysis of differentially expressed proteins (DEPs)

Based on the selection criteria of FC of > 1.2 or < 0.83 (*p* < 0.01), we detected 968 and 359 DEPs in the NC *vs*. ET and ET *vs*. CM comparisons, respectively. Specifically, 332 upregulated and 636 downregulated DEPs were identified in the NC vs. ET comparison, while 236 and 123 upregulated and downregulated proteins were detected in the ET *vs*. CM comparison, respectively (Table S6). In comparison, in the NC *vs*. ET and ET *vs*. CM groups, the pathways associated with inflammation and immune system were detected, such as inflammatory mediator regulation of the TRP channels, the IL-17 signaling pathway, the Toll-like receptor signaling pathway, inflammatory bowel disease, and Th17 cell differentiation, of which three DEPs (Hnf4a, Hsp90aa1, and IlL) were upregulated in the ET group, while downregulated in the CM group. Meanwhile, in comparison to the NC group, 13 DEGs (Stat5a, H2-Aa, Epas1, Esrra, H2-Ob, Fadd, Pias3, Pik3cb, Pik3ca, Cyp20a1, Pkn1, Bdnf, and Prkx) related to the immune system were upregulated in the CM group, while downregulated in the ET group (Fig. S6). Based on the functional analysis, 10 DEPs were identified to play potential roles in the protective effect of CM against chronic alcohol disease.

### Integrated analysis of transcriptome and proteome data

**Figure 7:**
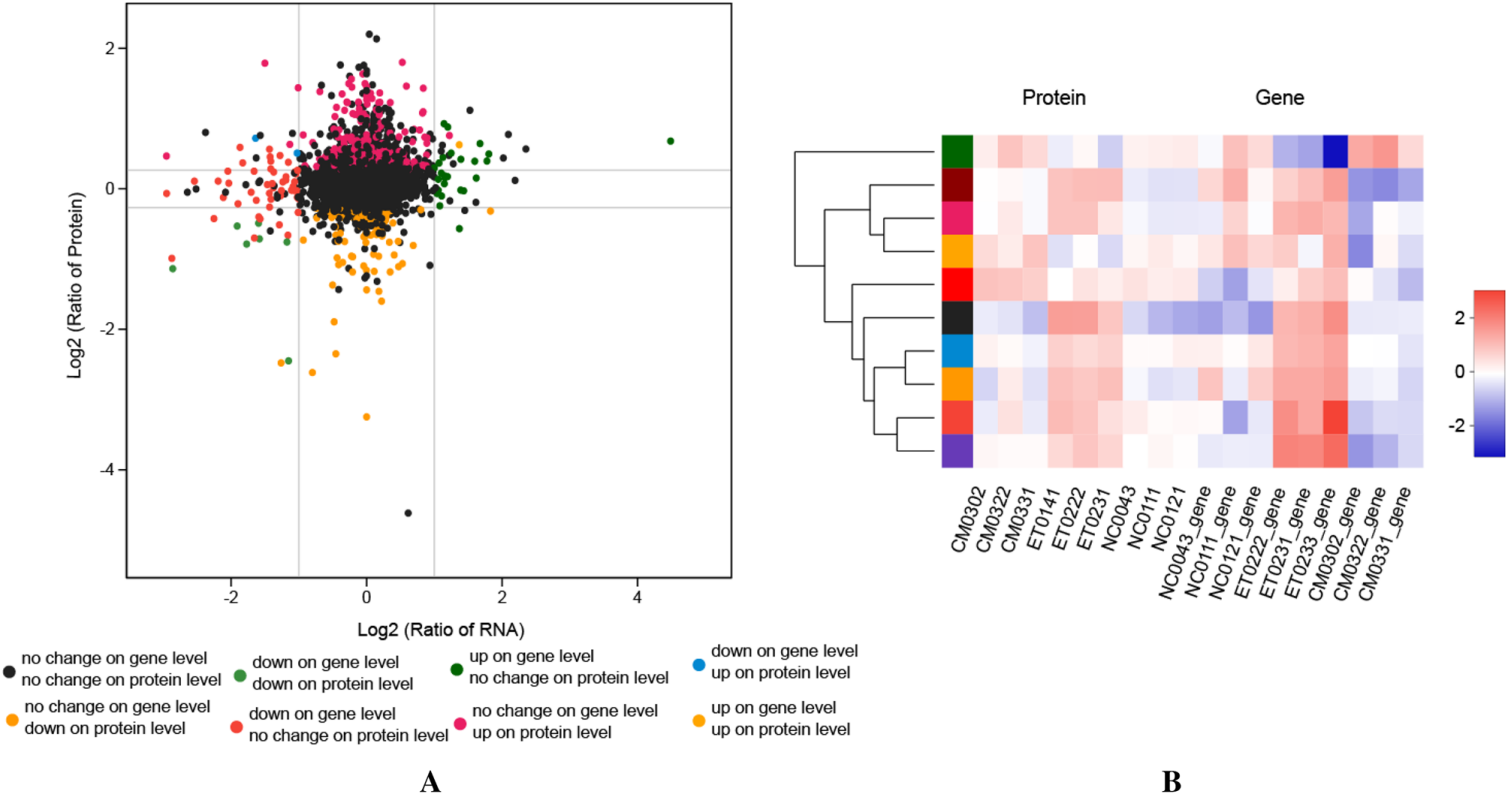
The Pearson correlation coefficient of the fold changes of ET/CM between the mRNA and protein expression levels (**A**), and the Pearson’s correlation coefficient of the fold change in the nine genes (**B**).

In our study, we explored the correlation between RNA and protein abundance in multiple ways to provide a global view of the RNA–protein correlation in the intervention of camel milk on alcoholic liver disease. Integrating the 11,702 detected genes by RNA-Seq and the 5621 proteins via TMT, 5318 genes showed expression values in both the mRNA and protein levels. Pearson’s correlation coefficients (0.0685, *p* < 0.01; Fig. 7A) were then calculated for each RNA–protein pair across all samples, showing that the majority of these pairs were positively correlated. To further understand the relationship between transcripts and proteins, we compared the intersection between the DEGs and DEPs. Most of the genes were significantly expressed at the mRNA level, but not at the protein level. At both the protein and mRNA levels, nine genes (Ppp1r3b, Mt1, Etnppl, St3gal5, Col4a2, Lpin1, Samd4, Lyve1, and Insig2) were found to overlap, and the Pearson’s correlation coefficient of the fold change in the nine genes was 0.6884 (Fig. 7B). Among them, *Etnppl* displayed great fold change (fold change in transcript, > 4; fold change in transcript, > 1.3) in the CM group (Table 2). PPP1R3B, especially, influences the accumulation of hepatic triglycerides, while ETNPPL can modulate the proliferation of hepatocellular cells. As a phosphatidic acid phosphatase (PAP) enzyme, LPIN1 plays an important role in lipid metabolism.

**Table 2:** Differentially expressed genes at both the mRNA and protein levels between ET and CM groups.

Gene	Transcript			Protein		
	Log_2_ Fold change	*p* value	Regulation type	Log_2_Fold change	*p* value	Regulation type
Ppp1r3b	− 1.2436	1.9491E−11	Down	− 0.4909	4.5680E−03	Down
Mt1	− 2.3489	8.4434E−31	Down	− 2.4526	8.3389E−03	Down
Etnppl	2.0425	2.7835E−04	Up	0.6261	4.049E−02	Up
St3gal5	− 2.6749	1.2795E−51	Down	− 0.5318	4.771E−03	Down
Col4a2	− 1.4117	5.0456E−18	Down	− 0.7589	6.667E−03	Down
Lpin1	− 3.1039	3.9587E−50	Down	− 1.1393	7.5000E−05	Down
Samd4	− 1.4088	3.5612E−06	Down	0.7194	6.6490E−03	Up
Lyve1	− 1.6445	9.6872E−26	Down	− 0.7139	3.4570E−03	Down
Insig2	− 2.6133	1.2400E−06	Down	− 0.7881	4.3840E−02	Down

## Discussion

A key problem in ALD research is the lack of animal models that can simulate the whole spectrum of human ALD. There are significant differences in the alcohol metabolism between rodents and humans, as well as great differences in the liver injury between the same and different strains of mice of different ages. At present, the most widely used alcoholic liver injury models are the acute alcohol gavage model, the Lieber–DeCarli model, the Gao binge model, and the Lieber DeCarli + Gao binge model (NIAAA model)^17^. These models represent the mild and early stages of human ALD. The NIAAA model belongs to the chronic-plus-binge alcohol feeding model in mice, which mimics the drinking pattern of patients with alcoholic hepatitis with a background of drinking for a number of years (chronic) and with a history of recent excessive alcohol consumption (binge)^18^. In the present study, the mouse were fed 4% Lieber DeCarli alcohol liquid feed for 8 weeks, and a high dose of alcohol (5 g/kg) was given by gavage at 7:00–9:00 a.m. at the beginning of week 9. The activities of ALT and AST in serum were significantly increased, and severe fatty lesions occurred in liver tissue in the ET group, indicating that the NIAAA model was successfully established. Meanwhile, compared to the ET group, camel milk can significantly reduce ALT and AST activity in mice serum and can improve their liver histology. This reveals that camel milk has a protective effect on the alcoholic liver injury induced by Lieber DeCarli alcohol liquid feed. This has been confirmed by previous research results in this laboratory^12^. Oxidative stress is one of the main pathogenesis of ALD. Long-term heavy drinking leads to a surge of ROS in the liver and the consumption of a large number of antioxidants (such as GSH and SOD), leading to an imbalance in the oxidation antioxidant balance and oxidative stress^19^. Excessive free radicals react with lipid to produce MDA, which further aggravates oxidative stress^20^. Our results show that the SOD activity and GSH content in the liver tissue of mice in the ET group significantly decreased, while the MDA content significantly increased, implying that a more serious lipid peroxidation reaction occurred in the mice in the ET group. After intervention with camel milk, the levels of SOD and GSH significantly increased, while MDA content significantly decreased. The results show that camel milk has an obvious protective effect on the lipid peroxidation induced by alcohol.

Studies have found that chronic alcohol intake accelerates steatosis by upregulating FAS and SCD1 activities^21,22^. Acetaldehyde, a metabolite of ethanol, can directly inhibit PPARa activity to promote steatosis. In addition, ROS and TNF-α can damage the secretion and transportation of triglycerides and can accelerate steatosis^23^. In this experiment, the content of TG in the livers of the ET group was higher than in that of the NC group. A large number of lipid droplets accumulated in the liver cells of the mice, resulting in severe steatosis. Moreover, the results of an inflammatory factor test once again confirmed that alcohol damages the secretion and transportation of triglycerides in hepatocytes. After camel milk intervention, the content of TG in hepatocytes was significantly reduced, the accumulation of lipid droplets in hepatocytes was significantly reduced, and the steatosis was improved.

Another mechanism of ALD is inflammation^6^. Acetaldehyde, a metabolite of alcohol, acts directly on the intestinal tract and increases the permeability of intestinal mucosa. In the ET group, the contents of TNF-α, IL-1β, IL-6, and IL-10 in the liver tissue significantly increased, while the content of IL-10 significantly decreased, which indicates that a large amount of alcohol intake increases the intestinal permeability and results in LPS translocating to the liver, thereby causing an inflammatory reaction. These results are consistent with those of Bertola et al.^24^ and Xia et al.^25^. After camel milk treatment, the contents of TNF-α, IL-1β, and IL-6 in the liver tissue of the mice significantly decreased, while the content of IL-10 significantly increased, indicating that camel milk can produce a large number of anti-inflammatory factors to inhibit the release of pro-inflammatory factors. In previous study of our team in 2012^12^, we also found that camel milk modulates liver inflammation caused by acute alcohol injury. In addition, the content of LPS in the serum of the mice decreased significantly in the CM group, which indicates that camel milk can prevent alcohol-induced colonic dysfunction and can inhibit an increase in the content of LPS in serum. Gut microbiota analysis showed that camel milk can regulate the intestinal flora of ALD mice. The main mechanism of improving ALD is to regulate the intestinal flora by changing the environment of said intestinal flora, and the resulting changes include promotion of the reproduction of intestinal dominant probiotics and inhibition of the growth of harmful intestinal bacteria.

Furthermore, we described transcriptomic and proteomic data sets to analyze, in a parallel and integrative manner, to decipher the pathological mechanisms of the intervention of camel milk in alcoholic liver disease. These analyses would enable us to identify dysregulated biological processes and signaling pathways at both the RNA and protein levels. Owing to the chronic ingestion of alcohol, lipid peroxidation occurs. Products of lipid peroxidation, such as malondialdehyde and 4-hydroxynonenal, result in protein adduct formation and serve as antigens to cause adaptive immunity^7^. Immune function is paradoxically enhanced by inflammation. In the current study, camel milk intervention significantly affected the inflammatory and immune system signaling pathway, as well as inhibited the production of pro-inflammatory factors and reduced the expression of immune genes, both at the transcriptomic and the proteomic levels (Figs. S5, S6).

Compared to cow milk, camel milk is well tolerated by lactose-deficient children with hypersensitivity due to^26^, and is consumed as an essential nutritional supplement with a high energy and vitamin content to help immune-deficient patients^27^. Besides its low content of fat, cholesterol, and lactose, along with its higher mineral, vitamin, and secretory IgA and IgM contents, camel milk contains several nano-antibodies with marked antibacterial and antiviral activities^28^. It also contains various bioactive proteins with immunomodulatory properties, including lysozymes, lactoperoxidase, and N-acetyl glucosa-minidase^28^. Moreover, CM is rich in lactoferrin, a protein with marked antioxidant and anti-inflammatory properties^29,30^. Therefore, camel milk has been consumed as an essential nutritional supplement with a high energy and vitamin content to help immune-deficient patients for a long time.

## Conclusions

Camel milk contains whey protein, lactoferrin, immunoglobulins, lysozyme, and vitamin C, and other nutrients, which considered to relate to anti-inflammatory, antioxidant, and immune system-regulating. At present, based on the pathogenesis of ALD, exploring functional foods related to anti-inflammatory and eliminating oxidative stress, and then antagonizing oxidative stress and inflammatory injury induced by alcohol metabolism, is considered to be an effective adjuvant strategy for the treatment of alcoholic liver injury. In this study, we attempted to investigate the effects of CM on the hepatic biochemical and multiple omics alterations induced by chronic alcoholic liver disease (ALD). As a result, camel milk not only prevents alcohol-induced colonic dysfunction and lipid accumulation, but also regulates oxidative stress and inflammatory cytokine production to protect against chronic ALD in mouse. Furthermore, camel milk can improve alcoholic liver injury in mice by regulating inflammatory factors and immune system disruptions.

## Materials and methods

### Ethics statement and animals

All experimental design and procedures of this study was carried out in compliance with the ARRIVE guidelines (https://arriveguidelines.org). In addition, the experimental procedures were performed by the National Institutes of Health Guidelines for the Care and Use of Laboratory Animals (Publication No. 85-23, revised 1985). Our research was supported by the Review Committee for the Use of Human or Animal Subjects of the Food Science and Engineering College of Inner Mongolia Agricultural University (Hohhot, China).

Male C57BL/6NCr mice (20 ± 2 g, 8–10 weeks old) were obtained from the Beijing Vital River Laboratory Animal Technology Co., Ltd (Beijing, China). The mice were housed in ventilated cages for a 12 h light/dark cycle at 20–25 ℃ and 50–60% relative humidity with free access to food and water ad libitum.

## Experimental groups and treatment protocol

After 1 week of acclimation, a total of 24 mice were randomly divided into the following three groups (*n* = 8 per group): Lieber DeCarli liquid diet control group (NC), ethanol-containing Lieber DeCarli liquid diet group (ET), and the camel milk group (CM). According to the research^31^, the Lieber DeCarli + Gao binge model (NIAAA model) was employed. The mice in the NC group were fed a Lieber DeCarli liquid (LDC) diet, while the ET group was fed an ethanol-containing LDC liquid diet (ethanol v/v accounted for 28% the total caloric intake) for 8 weeks. The CM group was given an ethanol-containing LDC liquid diet for the first four weeks, then an ethanol-containing LDC liquid diet with camel milk (3 g/kg body weight). On the first day of week 9, the ET and CM groups received 31.5% (v/v) ethanol by oral gavage at a dose of 7.3 g/kg, while NC group was given oral gavage of equal calorie maltodextrin solution (Fig. 1). After modeling, the animals were fasted for 9 h and anesthetized with isoflurane gas. Aortic blood, feces samples and liver tissues were collected and stored at − 80 °C. Bactrian camel milk (CM) was collected from a private pasture in Bayan Nur City, Inner Mongolia, China, and transported to the laboratory for further use. Skimmed camel milk was obtained with reference to previous research^12^.

## Determination of serum/liver biochemical indicators

### Alanine transaminas (ALT) and aspartate transaminase (AST) analysis

Liver cell damage can lead to increased liver function enzyme activities, which can sensitively reflect the extent of liver injury^32^. Serum was isolated by centrifugation (2000×*g* for 15 min at 4 ℃). The levels of ALT and AST were determined using commercially available kits (MAK052 and MAK055, respectively; Sigma-Aldrich, St. Louis, MO, USA) according to the manufacturer’s instructions.

### Malondialdehyde (MDA), superoxide dismutase (SOD), and glutathione (GSH) analysis

Samples of liver were homogenized for 15 s at 3.10 m/s in PBS (pH 7.4) and then centrifuged (3000×*g* at 4℃ for 20 min). Supernatant was collected, and the concentrations of MDA, SOD, and GSH were determined using the relevant ELISA kits (Shanghai Enzyme-Linked Biotechnology Co. Ltd., Shanghai, China) according to the manufacturer’s instructions.

### Liver tissue plasma inflammatory factors analysis

The levels of tumor necrosis factor-a (TNF-a), IL-6, IL-1β, and IL-10 were detected using ELISA kits (ml002095, ml063159, ml063132, and ml037873 respectively; ML Bio Solutions, Charlotte, NC, USA) according to the manufacturer’s protocol.

### Lipopolysaccharide (LPS) and triglyceride (TG) analysis

LPS is an endotoxin that can activate hepatic Kupffer cells. It is also an important factor in activating liver damage, such as inflammatory response and apoptosis^31^. The level of LPS in serum was measured using ELISA kits (Shanghai Enzyme-Linked Biotechnology Co. Ltd.) according to the manufacturer’s protocols. The level of TG (triglyceride) in the liver tissue was measured using ELISA kits (Shanghai Enzyme-Linked Biotechnology Co. Ltd.) according to the manufacturer’s protocols.

### Gut microbiota analyses

Genomic DNA was extracted from feces using an E.Z.N.A.^®^ Soil DNA kit (Omega Bio-tek, USA). The V3 and V4 regions of the 16S rRNA genes were amplified using general primers (338F/806R). A Phusion High-Fidelity PCR Master Mix Kit (catalog no. M0541, New England Bio labs, Ipswich, MA) was used to carry out all of the PCR reactions, and purified using the FastPfu Polymerase AxyPrep DNA Gel Extraction Kit (Axygen Biosciences, Union City, CA). The MiSeq PE (pair end) library was constructed using the NEXTFLEX Rapid DNA-Seq Kit (Bioo Scientific, USA), and were sequenced using the MiSeq system (MiSeq PE 300, Illumina, San Diego, CA, USA) at the Shanghai Majorbio Bio-Pharm Technology Co. Using QILME version 1.9.1, the sequences were further demultiplexed and analyzed. Operational taxonomic unit (OTU) clustering was performed at 97% sequence identity.

### Transcriptomic analysis

#### RNA extraction and sequencing

RNA sequencing was performed on 9 liver samples (NC0043, NC0111, and NC0121 from the NC group; ET0222, ET0231, and ET0233 from ET group; CM0302, CM0322, and CM0331 from CM group). Based on the research of Ming et al. (2020), we completed total RNA extraction and evaluated its integrity, concentration, and purity of each sample. The transcriptome library was constructed using the TruSeqTM RNA sample preparation kit (Illumina, San Diego, CA, USA) according to the manufacturers’ instructions, and the libraries were sequenced on the HiSeq 4000 ultra-high-throughput sequencing system.

#### Mapping and annotation

After removal of the sequencing adapters, low-quality reads, and reads containing ploy-N, the remaining clean reads were aligned to the whole mouse genome (*Mus_musculus*, GRCm38.p6). The clean reads were mapped to the reference genome of *Mus_musculus* (version, GRCm38.p6) using TopHat2 software (version 2.1.1)^33^. Quality control and read statistics were determined using FastQC software.

#### Quantification and differential gene analysis

Fragments per kilobases per million reads (FPKM) values obtained using Cufflink software (version 2.1.1) were used as the values for normalized gene expression^34^. Differentially expressed genes (DEGs) of three groups (NC vs. ET, ET vs. CM, and NC vs. CM) were performed using the edge R package (version, 3.5) based on *p* < 0.05 and |log_2_ fold change|≥ 2. The resulting *p-*values were adjusted using the Benjamini–Hochberg method for controlling the false discovery rate (FDR)^35^. Using the relevant databases, Gene ontology (GO) and Kyoto Encyclopedia of Genes and Genomes (KEGG) pathway enrichment analyses (the citation guidelines: www.kegg.jp/kegg/kegg1.html) of the DEGs were performed.

### Proteomics analysis

#### Protein extraction, digestion and TMT labeling

Proteomic analysis was performed on 9 liver samples (the analyzed samples were the same as the transcriptome). Total protein was extracted from the liver tissue of the mice using a urea lysis buffer with a protease inhibitor. Protein concentrations and quantification were performed by the BCA Protein Assay Kit (Pierce, Thermo, USA) protocol. Protein digestion was completed according to the standard procedure, and the resulting peptide mixture was labeled using the 10-plex TMT reagent (Thermo fisher, Art.No.90111) according to the manufacturer’s instructions (Thermo Fisher Scientific).

#### High pH RPLC separation and LC–MS/MS analysis

The pooled samples were fractionated into fractions by ACQUITY Ultra Performance liquid chromatography (Waters, USA) with an ACQUITY UPLC BEH C18 Column (1.7 µm, 2.1 × 150 mm, Waters, USA) to increase the proteomic depth. Labeled peptides were analyzed by online nano-flow liquid chromatography tandem mass spectrometry performed on a Q Exactive Plus quadrupole orbitrap mass spectrometer (Thermo, USA) through a nano-electrospray ion source.

#### Protein identification and quantification

The RAW data files were analyzed using ProteomeDiscoverer (Thermo Scientific, Version 2.2) against the Mus_musculus database (http://asia.ensembl.org/Mus_musculus/Info/Index, Assembly Version GRCm38, 67856 s). The MS/MS search criteria were as follows: Mass tolerance of 10 ppm for MS and 0.02 Da for MS/MS Tolorance, trypsin as the enzyme with two missed cleavages allowed, carbamido methylation of cysteine and the TMT of N-terminus and lysine side chains of peptides as fixed modification, and methionine oxidation as dynamic modifications, respectively. The false discovery rate (FDR) of the peptide identification was set as FDR ≤ 0.01. A minimum of one unique peptide identification was used to support protein identification. Relative quantification of the identified proteins was estimated according to the weighted and normalized ratios of uniquely identified peptides using the median ratio in Mascot^36^. Proteins with significant differences in expression between pairs of groups and with expression ratio thresholds of ≥ 1.2-fold increase or ≤ 0.83-fold decrease were identified. Differentially expressed proteins (DEPs) were further used for GO and KEGG enrichment analysis. Protein–protein interaction analysis was performed using String v10.5.

#### Statistical analysis

All data were presented as the mean ± standard error of the mean (SEM) using SPSS (17.0). Differences between groups were evaluated by one-way analysis of variance (ANOVA) followed by Tukey’s post-hoc multiple comparison tests using GraphPad Prism software (Version 7). Bioinformatics data analysis was performed using R software. *p* < 0.05 was considered to indicate statistical significance (significantly different from the control group at *p < 0.05 and **p < 0.001; significantly different from the model group at ^#^*p* < 0. 05 and ^##^*p* < 0.001).

## Supplementary Information

Supplementary Information.
