# Supplementary material for: Camel milk ameliorates inflammatory mechanisms in an alcohol-induced liver injury mouse model
Source: Sci Rep. 2021 Nov 24;11:22811. doi: 10.1038/s41598-021-02357-1 (PMC8613211; doi:10.1038/s41598-021-02357-1)

**Table S1.** Effects of CM on the levels of MDA, SOD, and GSH in mouse liver.

| **Groups** | **MDA (nmol/g)** | **SOD (U/mg)** | **GSH (ng/g)** |
| --- | --- | --- | --- |
| NC | 1.34±0.09 | 1184.41±41.81 | 10.03±0.85 |
| ET | 1.86±0.18^*^ | 1079.49±58.45 | 6.95±1.36 |
| CM | 1.57±0.07 | 1026.43±29.89 | 24.90±1.70^##^ |

NC: control group; ET: ethanol-containing Lieber–DeCarli liquid diet group; CM: camel milk group. Data represent the mean ± standard error of the mean (n = 8 mice). Significantly different from the control group at **P* < 0. 05 and ***P* < 0.001; Significantly different from the ET group at #*P* < 0. 05 and ##*P* < 0.001.

**Table S2**. Number of valid sequences, operational taxonomic units (OTUs), diversity, richness, and estimated sample coverage in each sample.

| **Sample ID** | **Valid sequences** | **OTUs** | **Chao1** | **Shannon** | **Coverage/%** |
| --- | --- | --- | --- | --- | --- |
| NC0032 | 28970 | 241 | 283.27 | 3.66 | 0.9982 |
| NC0043 | 35046 | 225 | 277.14 | 3.54 | 0.9980 |
| NC0111 | 39746 | 241 | 250.03 | 3.52 | 0.9987 |
| NC0112 | 23063 | 241 | 266.62 | 3.73 | 0.9984 |
| NC0113 | 30777 | 262 | 277.50 | 3.92 | 0.9986 |
| NC0121 | 26793 | 246 | 287.58 | 3.62 | 0.9979 |
| ET0132 | 37331 | 214 | 239.79 | 2.52 | 0.9985 |
| ET0202 | 32256 | 216 | 242.64 | 2.67 | 0.9984 |
| ET0212 | 39835 | 225 | 272.59 | 3.29 | 0.9983 |
| ET0222 | 28343 | 229 | 266.13 | 2.82 | 0.9980 |
| ET0231 | 34157 | 192 | 234.27 | 2.79 | 0.9982 |
| ET0233 | 39306 | 209 | 274.05 | 2.39 | 0.9977 |
| CM0302 | 27399 | 252 | 275.37 | 2.94 | 0.9979 |
| CM0311 | 34716 | 249 | 322.23 | 3.58 | 0.9976 |
| CM0312 | 35400 | 226 | 274.26 | 3.17 | 0.9978 |
| CM0321 | 38741 | 259 | 299.23 | 3.21 | 0.9979 |
| CM0322 | 35937 | 230 | 308.11 | 3.42 | 0.9977 |
| CM0331 | 30080 | 261 | 251.50 | 2.91 | 0.9983 |

**Table S3.** Summary of the sequencing reads alignment to the Mus_musculus (GRCm38.p6) reference genome.

| Sample | Clean reads | Clean bases | Q20(%) | Q30(%) | GC content | Mapped reads | Proportion | Multiple mapped | Uniquely mapped |
| --- | --- | --- | --- | --- | --- | --- | --- | --- | --- |
| NC0043 | 51622830 | 7690310549 | 99.01 | 96.64 | 49.96 | 49151088 | 95.21% | 3899809(7.55%) | 45251279(87.66%) |
| NC0111 | 48605476 | 7246087416 | 99.04 | 96.69 | 49.85 | 46488041 | 95.64% | 3943856(8.11%) | 42544185(87.53%) |
| NC0121 | 46833114 | 6975498742 | 99 | 96.62 | 50.06 | 44838244 | 95.74% | 3469096(7.41%) | 41369148(88.33%) |
| ET0222 | 58806638 | 8794888044 | 99.07 | 96.74 | 49.78 | 55801429 | 94.89% | 5872889(9.99%) | 49928540(84.9%) |
| ET0231 | 58436922 | 8719539333 | 99.05 | 96.70 | 49.22 | 54455742 | 93.19% | 7019779(12.01%) | 47435963(81.17%) |
| ET0233 | 56513354 | 8411144275 | 98.99 | 96.52 | 49.78 | 53314662 | 94.34% | 6227599(11.02%) | 47087063(83.32%) |
| CM0302 | 55130352 | 8203379842 | 98.96 | 96.44 | 49.76 | 52426609 | 95.10% | 4995926(9.06%) | 47430683(86.03%) |
| CM0322 | 57159436 | 8513573943 | 99.04 | 96.68 | 49.63 | 53950940 | 94.39% | 5684542(9.95%) | 48266398(84.44%) |
| CM0331 | 51745564 | 7701882702 | 98.97 | 96.54 | 49.90 | 49346252 | 95.36% | 4438655(8.58%) | 44907597(86.79%) |

**Figure S1.** Relative abundance bacterial species in the gut microbiota of mice in the three treatment groups at the family (**A**) and genus (**B**) levels.


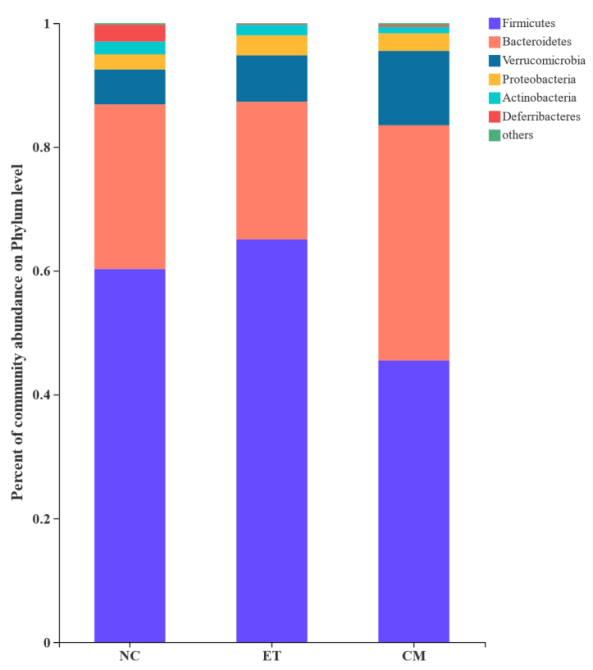

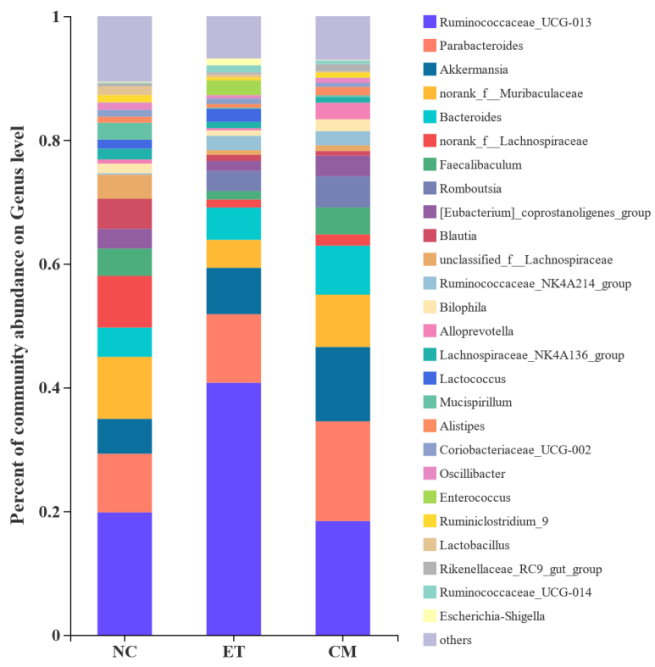


**(B)**

**(A)**

**Figure S2.** Abundance of bacteria taxa at the genus levels.


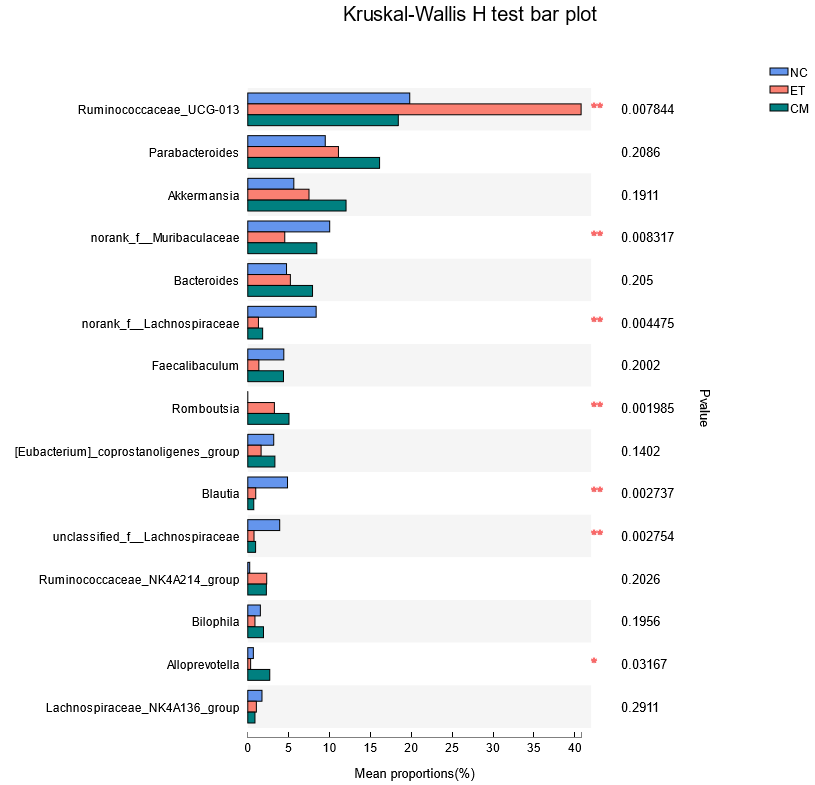


**Figure S3.** Differences in species composition at the genus level. Species composition differences between (**A**) the ET and CM groups and (**B**) the ET and NC groups.


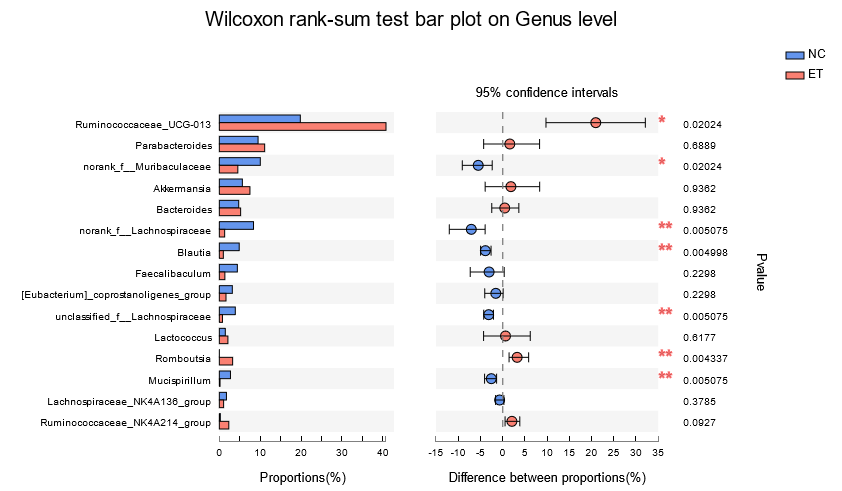


**(A)**


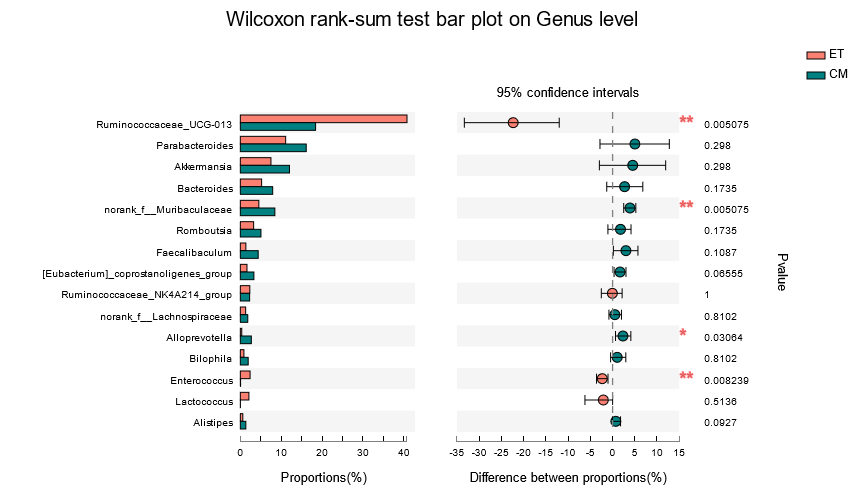


**(B)**

**Figure S4.** Venn diagram showing (**A**) gene counts expressed in the NC, ET, and CM groups and (**B**) comparative analysis of the number of DEGs among different groups.

| 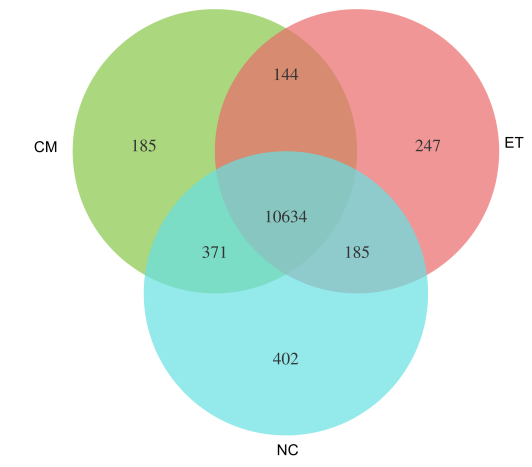 | 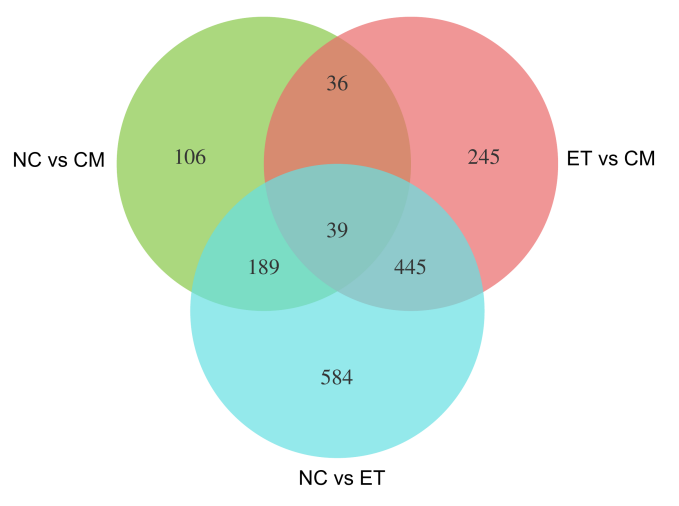 |
| --- | --- |
| (**A**) | (**B**) |

**Figure S5** Expression of selected related to inflammatory, and immune system genes based on RNA-Seq.


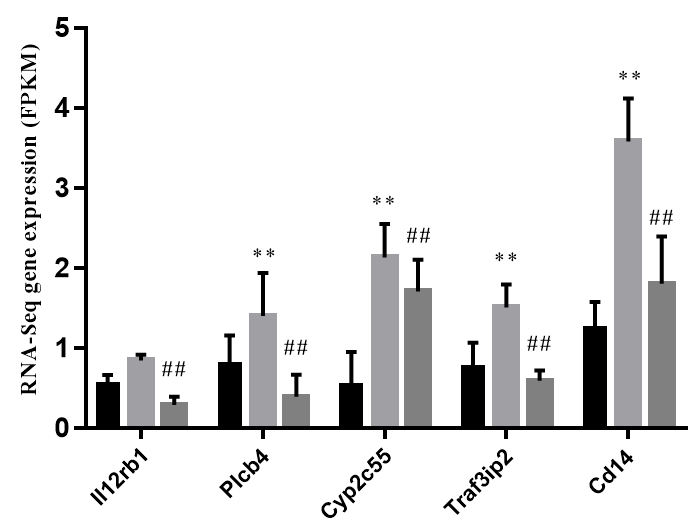

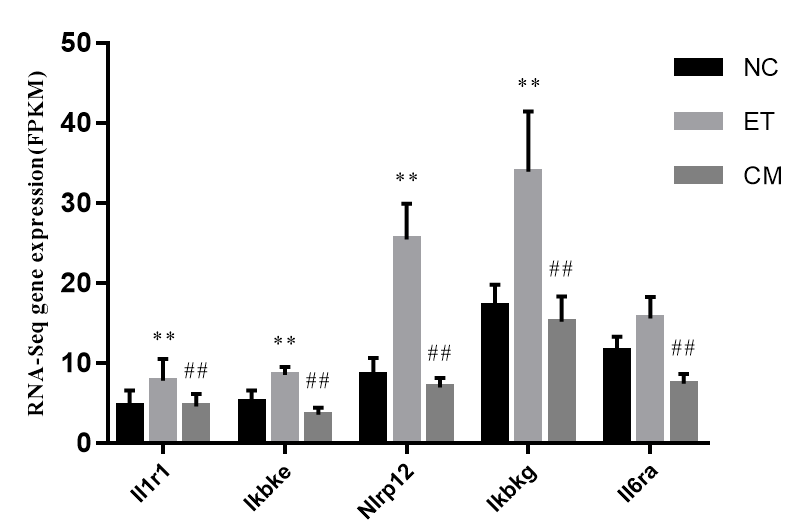


**Figure S6** Expression of selected related to inflammatory, and immune system proteins based on LC-MS/MS.


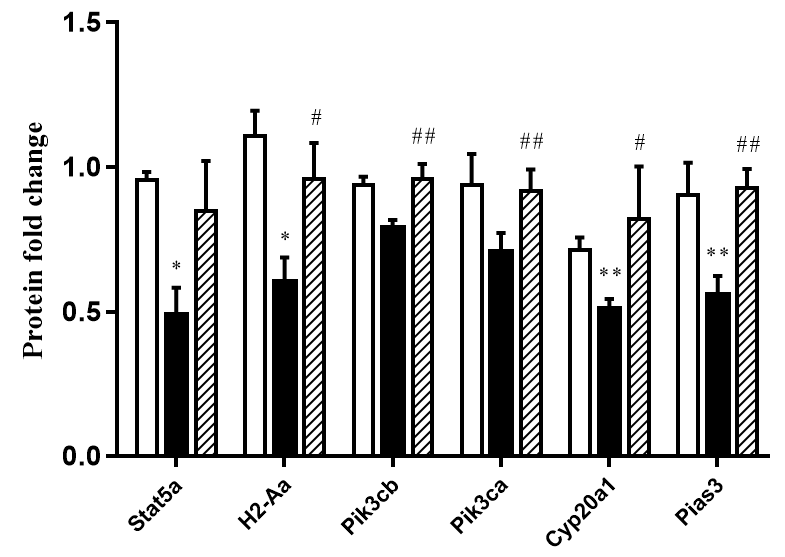

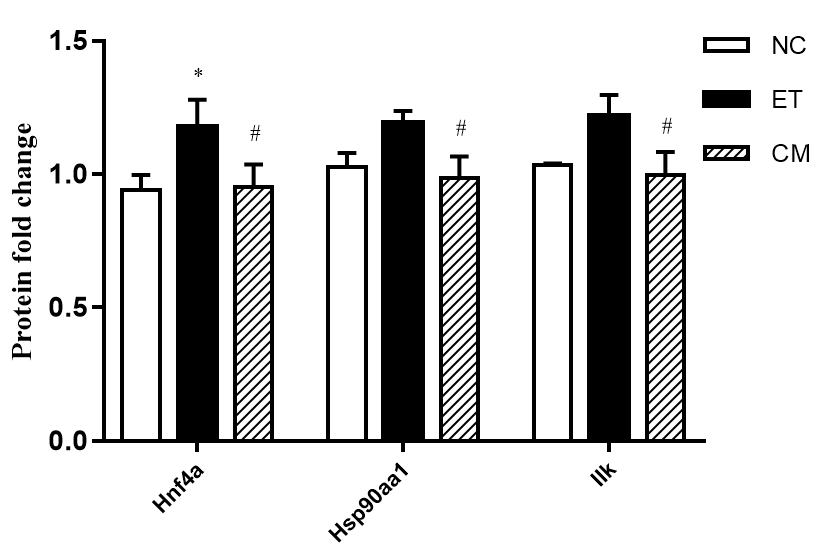

Supplement: Supplementary file 1 — Supplementary Information. [file 41598_2021_2357_MOESM1_ESM.zip › Supplementary materials/Supplementary.docx]
